# Anti-mold, self-cleaning superhydrophobic bamboo fiber/polypropylene composites with mechanical durability

**DOI:** 10.3389/fchem.2023.1150635

**Published:** 2023-03-21

**Authors:** He Zhao, Xinxing Lin, Shengchang Lu, Hui Wu, Xiaxing Zhou, Liulian Huang, Jianguo Li, Jianping Shi, Wenxuan Tong, Hongmei Yuan, Lihui Chen

**Affiliations:** ^1^ College of Material Engineering, Fujian Agriculture and Forestry University, Fuzhou, Fujian, China; ^2^ National Forestry and Grassland Administration Key Laboratory of Plant Fiber Functional Materials, Fuzhou, Fujian, China; ^3^ College of Materials and Environmental Engineering, Fujian Polytechnic Normal University, Fuzhou, Fujian, China; ^4^ School of Forestry, Henan Agricultural University, Zhengzhou, China

**Keywords:** bamboo fiber/polypropylene composites, superhydrophobic, anti-mold, self-cleaning, mechanical durability

## Abstract

Bamboo fiber/polypropylene composites (BPCs) have been widely used in buildings, interior decoration, and automobile components. However, pollutants and fungi can interact with the hydrophilic bamboo fibers on the surface of Bamboo fiber/polypropylene composites, degrading their appearance and mechanical properties. To improve their anti-fouling and anti-mildew properties, a superhydrophobic modified Bamboo fiber/polypropylene composite (BPC-TiO_2_-F) was fabricated by introducing titanium dioxide (TiO_2_) and poly(DOPAm-*co*-PFOEA) onto the surface of a Bamboo fiber/polypropylene composite. The morphology of BPC-TiO_2_-F was analyzed by XPS, FTIR, and SEM. The results showed that TiO_2_ particles covered on Bamboo fiber/polypropylene composite surface *via* complexation between phenolic hydroxyl groups and Ti atoms. Low-surface-energy fluorine-containing poly(DOPAm-*co*-PFOEA) was introduced onto the Bamboo fiber/polypropylene composite surface, forming a rough micro/nanostructure that endowed BPC-TiO_2_-F with superhydrophobicity (water contact angle = 151.0° ± 0.5°). The modified Bamboo fiber/polypropylene composite exhibited excellent self-cleaning properties, and a model contaminant, Fe_3_O_4_ powder, was rapidly removed from the surface by water drops. BPC-TiO_2_-F showed excellent anti-mold performance, and no mold was on its surface after 28 days. The superhydrophobic BPC-TiO_2_-F had good mechanical durability and could withstand sandpaper abrasion with a weight load of 50 g, finger wiping for 20 cycles, and tape adhesion abrasion for 40 cycles. BPC-TiO_2_-F showed good self-cleaning properties, mildew resistance, and mechanical resistance, giving it promising applications for automotive upholstery and building decoration.

## 1 Introduction

Biobased plastic composites formed by blending biomass resources with thermoplastics have attracted attention for applications in buildings, decoration, automotive interiors, and decking (Bengtsson et al., 2007; Kuo et al., 2009; Durmaz et al., 2021; Guo et al., 2021) because of their broadly-distributed resources, low cost, lightweight, high toughness, sound insulation, and shock absorption properties (Rowell, 2007; Kuo et al., 2009; Ren et al., 2020; Vedat and Fatih, 2020). Bamboo fiber (BF) is an important biomass resource, with rapid renewability, wide availability, biodegradability, and outstanding mechanical properties (Yu et al., 2014). The tensile strength and Young’s modulus of bamboo fibers can reach as high as 1.43–1.93 GPa and 25.5–46.3 GPa, which are much higher than those of most other plant fibers, such as wood, flax, jute, and sisal (Yu et al., 2011; Sobczak et al., 2012). BF is a good substitute for wood fiber, carbon fiber, and glass fiber for preparing biomass plastic composites. Therefore, to add value to BF and reduce the use of petroleum-based plastics, bamboo fiber/polypropylene composites (BPCs) have been extensively used in various fields, such as buildings, interior decoration, and automobile components (Fei et al., 2016; Wang et al., 2019; Jamaludina et al., 2020). Therefore, BPCs need to withstand damage by water, mildew fungi, and mechanical wear during use.

Large amounts of hydrophilic fibers are exposed on the BPC surface, although most BFs are entrapped by polypropylene (PP). Those fibers are hygroscopic and rich in proteins, sugars, starches, and other nutrients (Liese, 1987; Shalbafan et al., 2013), which lead to contamination by mold and fungi that may reduce mechanical properties and accelerate aging. Therefore, it is necessary to modify BPCs to achieve hydrophobicity and self-cleaning properties to prolong their service life.

Numerous studies have shown that physical and chemical modification of bamboo fibers or plastic matrixes can improve the performance of BPCs; however, physical treatment methods generally consume large amounts of energy, while chemical modification processes usually produce toxic wastewater (Zhang et al., 2018; Sanjay et al., 2019). Therefore, it is better to adopt a method that consumes less energy, less wastewater, and can easily form mildew-proof, superhydrophobic, water-resistant, and self-cleaning layers on the surface of BPCs. On superhydrophobic surfaces with a water contact angle (CA) higher than 150° (Wang S. et al., 2011; Budunoglu et al., 2011; Wang et al., 2012; Nguyen-Tri et al., 2019), pollutants or dust can be easily removed by the rolling of water droplets. Generally, superhydrophobic surfaces can be obtained by a combination of rough surfaces and low-surface-energy substances (Windeisen et al., 2007; Xu and Cai, 2008; Yang and Deng, 2008; Zimmermann et al., 2008; Wang S. L. et al., 2011; Ellinas et al., 2011; Lu et al., 2015; Lin et al., 2016; Mrad et al., 2018; Wu et al., 2018; Li et al., 2020; Lu et al., 2022). It is still challenging to produce BPCs with anti-mold, self-cleaning, and mechanical durability through a simple and convenient process.

In this paper, we aimed to fabricate superhydrophobic, self-cleaning, and anti-mold BPCs as well as mechanical durability *via* a facile immersion method that introduced titanium dioxide on the BPC surface *via* hydrogen bonding. The low surface energy of rough micro/nanostructured copolymers with fluorinated units imparted the BPC with superhydrophobicity. The modified BPC exhibited excellent self-cleaning and anti-mildew properties with an easy-to-operate approach, showing great potential for applications in automotive upholstery and building decoration.

## 2 Materials and methods

### 2.1 Materials

Bamboo fiber (BF) was prepared by alkali pretreatment at a low temperature in a laboratory. The average length and width of BF were 25.8 mm and 175.0 μm, respectively. Polypropylene (PP) with a density of 0.91 g/cm^3^ and melting temperature of 165 °C was purchased from Qingdao Guangshengyuan Packaging Material Co., Ltd., China. *N*-(3,4-Dihydroxyphenethyl) acrylamide (DOPAm) and 97 wt% 2-(perfluorooctyl)ethyl acrylate (PFOEA) were supplied by Shanghai Aladdin Reagent Co., Ltd., China. Azobisisobutyronitrile (AIBN), titanyl oxysulfate, *N, N*-dimethylformamide (DMF), pyridine, methanol, ethanol, acetic acid, and ferroferric oxide (Fe_3_O_4_) were obtained from Shanghai Aladdin Reagent Co., Ltd., China. AK-225 was purchased from Guangzhou Guangwan New Material Co., Ltd., China. Potato dextrose agar (PDA) was obtained from Qingdao Hope Bio-Technology Co., Ltd., China.

Poly(DOPAm-*co*-PFOEA) was synthesized using DOPAm and PFOEA (Lin et al., 2016; Lu et al., 2021). DOPAm (0.08 g) was dissolved in 2 mL DMF. Then, 0.008 g AIBN and 1 mL FOEMA were added. Four freeze-thaw cycles were performed to remove O_2_. After reacting at 70°C for 6 h, the solution was dropped into methanol to produce a white precipitate. Poly(DOPAm-*co*-PFOEA) was obtained after the precipitate was dried under a vacuum for 24 h.

### 2.2 Preparation of bamboo fiber/polypropylene composites (BPCs)

BF and PP were alternately put into a mold (200 mm × 200 mm × 4 mm) using a layer-by-layer method. The weight ratio of BF and PP in BPC was 1: 1. A BPC with a density of 0.85 g/cm^3^ was obtained by pressing the specimen at 190°C under a pressure of 8 MPa for 20 min.

### 2.3 Modification of bamboo fiber/polypropylene composites (BPCs)

Titanyl oxysulfate (0.8 g) was added to 30 mL of deionized water. The pH of the solution was adjusted to 6 using 0.3 mol/L acetic acid solution. BPC was immersed in the solution and stirred at 70°C for 4 h. After the reaction, the composite was dried under a vacuum at 60°C for 24 h to obtain TiO_2_-modified BPC (BPC-TiO_2_). Then, BPC-TiO_2_ was soaked in 15 mL AK-225 solution containing 3.3 mg/mL poly(DOPAm-*co*-PFOEA) for 24 h and dried under vacuum at 60°C for 12 h to obtain superhydrophobic BPC (BPC-TiO_2_-F).

### 2.4 Characterization

X-ray photoelectron spectroscopy (XPS) was conducted using a Thermo Scientific ESCALAB 250Xi instrument (Waltham, MA, United States) with a standard Al-Kα X-ray source. The energy resolution of the spectrometer was set to a pass energy of 30.0 eV for survey scans at 0.05 eV per step and a 150.0 eV pass energy for region scans at 1.0 eV per step. The X-ray beam operated at an acceleration voltage of 13 kV and a current of 25 mA. Spectra were calibrated to the C 1 s peak at 285.0 eV.

Attenuated total reflectance Fourier-transform infrared (ATR-FTIR) spectroscopy was performed on a Perkin-Elmer Spectrum 2000 FTIR spectrometer (Norwalk, CT, United States), which was equipped with an MKII Golden Gate, single reflection ATR System from Specac Ltd., London, United Kingdom. BPC, BPC-TiO_2_, and BPC-TiO_2_-F were pressed against the ATR crystal to obtain a sufficient signal. The spectra were measured in the wavenumber range of 4,000–600 cm^-1^ at a resolution of 4 cm^-1^ with 32 scans.

The surface morphologies of the composites before and after modification were observed using a Hitachi S-4800 field emission scanning electron microscope (FESEM) from Tokyo, Japan. To enhance the sample conductivity before measurements, galvanic platinum deposition (5 mA) was conducted by spraying the samples for 110 s.

The wettability of BPC and BPC-TiO_2_-F was determined by a water drop shape analyzer of KRUSS DSA 30 from Germany. The contact angle (CA) was tested by dropping a 2 μL water droplet on the surface of BPC and BPC-TiO_2_-F. Five different locations on the same sample were recorded.

### 2.5 Anti-mold test

*Aspergillus niger* was used to determine the anti-mold performance of the composites. PDA powder (46.0 g) was dissolved in 1,000 mL of distilled water. After autoclaving at 115°C for 20 min, the PDA solution was poured into a Petri dish sterilized by ultraviolet light. After cooling, the activated *A. niger* was added into the culture medium using an inoculating needle and incubated at 28°C and a relative humidity of 85% for 7 days. The sterilized BPC and BPC-TiO_2_-F were put into a culture medium. Images of the samples were captured every day using a digital camera (Nikon, Japan).

## 3 Results and discussion

### 3.1 XPS analysis

**FIGURE 1:**
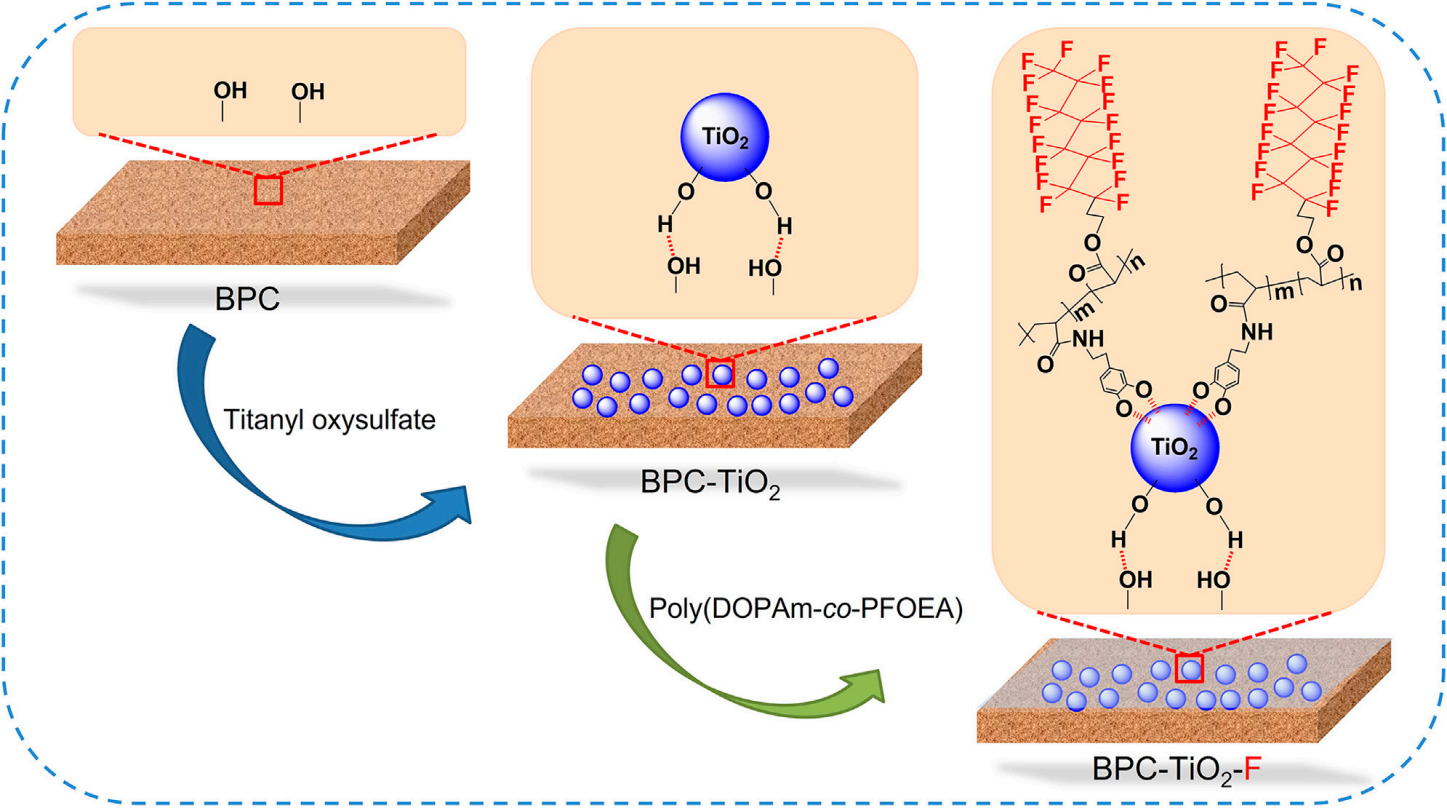
Schematic of surface modification of BPC by TiO_2_ particles and poly(DOPAm-*co*-PFOEA).

A schematic of the surface-modified BPC by TiO_2_ particles and poly(DOPAm-*co*-PFOEA) is shown in Figure 1. TiO_2_ particles were loaded *in situ* on the surface of BPC *via* hydrogen bonding (Guvendiren et al., 2008; Mahadik et al., 2010; Xu et al., 2012) with the hydroxyl groups of BF (Lu et al., 2014). Poly(DOPAm-*co*-PFOEA) was deposited on the surface of TiO_2_ particles through catechol-Ti complexation (Zeng et al., 2010). The catechol groups in DOPAm bonded to the TiO_2_ surface. The low-surface-energy fluorinated units in PFOEA covered the TiO_2_ particle exterior. Thus, a rough micro/nanostructure was created on the BPC surface, which decreased the surface energy of the composites and endowed the BPC with superhydrophobicity (Lin et al., 2016). After modification, the appearance of BPC was unchanged.

**FIGURE 2:**
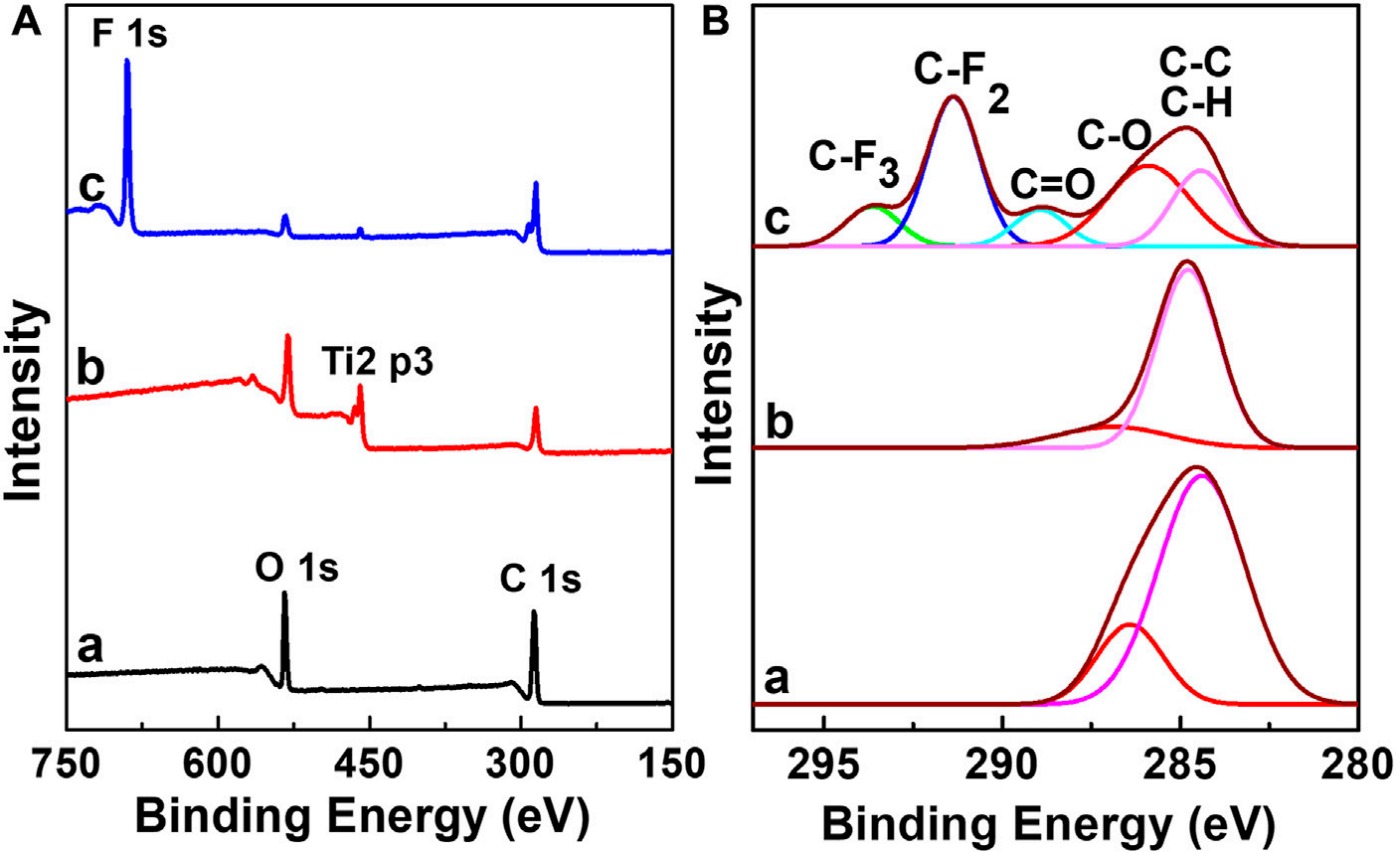
**(A)** Wide-scan and **(B)** C 1s narrow-scan XPS spectra of **(a)** BPC, **(b)** BPC-TiO_2_, and **(c)** BPC-TiO_2_-F.

Figure 2 shows the wide-scan and C 1s narrow-scan XPS spectra of BPC, BPC-TiO_2_, and BPC-TiO_2_-F. The peaks at binding energies of 533.0 eV and 284.8 eV were attributed to O 1 s and C 1s (Li et al., 2015) in the BPC, as shown in Figure 2A, a. After TiO_2_ particle deposition, a new peak of Ti 2p_3/2_ at a binding energy of 459.1 eV appeared, indicating that TiO_2_ particles were successfully coated on the surface of BPC by *in situ* synthesis, as shown in Figure 2A, b. After BPC-TiO_2_ was modified by poly(DOPAm-*co*-PFOEA), a new F 1 s peak appeared at 690.1 eV, indicating that poly(DOPAm-*co*-PFOEA) was successfully introduced onto the surface of BPC-TiO_2_, as shown in Figure 2A, c.

As shown in the narrow-scan C 1 s spectrum of untreated BPC in Figure 2A, b, the peaks at binding energies of 284.8 eV and 286.4 eV were attributed to C-H/C-C and C-O, respectively. After BPC-TiO_2_ was modified by poly(DOPAm-*co*-PFOEA), the C 1 s spectrum produced two new peaks at binding energies of 289.3 eV, 291.2 eV, and 293.4 eV, which belonged to the characteristic peaks of C=O, C-F_2_, and C-F_3_ (Baidya et al., 2017) of poly(DOPAm-*co*-PFOEA), as shown in Figure 2B, c.

Changes in the atomic composition of the composites were analyzed by XPS, as shown in Table 1. Compared with BPC, BPC-TiO_2_ showed a Ti content of 14.8% on the surface. The O/C ratio increased from 0.38 to 0.84. After being modified with poly(DOPAm-*co*-PFOEA), the Ti content in BPC-TiO_2_-F decreased to 0.7, while the F content increased to 48.9%, and the O/C ratio decreased to 0.17. These changes indicated that the surface of BPC was modified by poly(DOPAm-*co*-PFOEA).

**TABLE 1:** Atomic composition changes of BPC, BPC-TiO_2_, and BPC-TiO_2_-F.

Sample type	C(%)	O(%)	Ti(%)	F(%)	O/C ratio	F/C ratio
BPC	72.6	27.4			0.38	
BPC-TiO_2_	46.2	39.0	14.8		0.84	
BPC-TiO_2_-F	43.0	7.3	0.7	48.9	0.17	1.14

### 3.2 ATR-FTIR spectrometry

**FIGURE 3:**
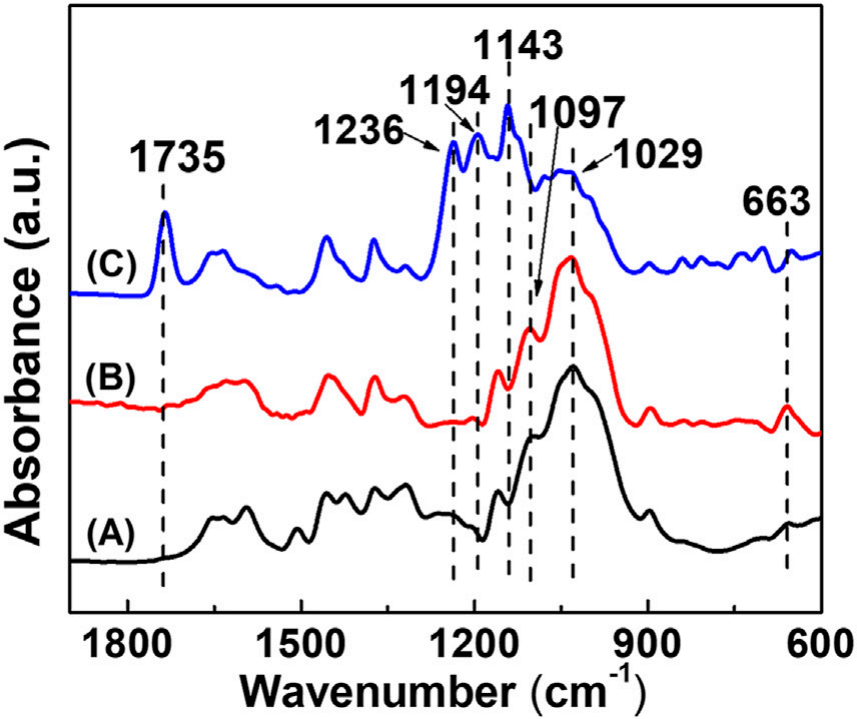
ATR-FTIR spectra of **(A)** BPC, **(B)** BPC-TiO_2,_ and **(C)** BPC-TiO_2_-F.

The ATR-FTIR spectra of the BPC surface modified by TiO_2_ particles and poly(DOPAm-*co*-PFOEA) are shown in Figure 3. The absorption peak at 1,033 cm^-1^ was the C-O stretching vibration peak of the BF skeleton. This peak changed little after TiO_2_ modification but weakened after being treated by poly(DOPAm-*co*-PFOEA). For BPC-TiO_2_-F, a new absorption peak at 1735 cm^-1^ appeared, which was the stretching vibration peak of C=O groups. The absorption peaks at 1,148 cm^-1^, 1,203 cm^-1^, and 1,236 cm^-1^ were associated with various stretching modes of -CF_2_ and -CF_3_ groups (Baidya et al., 2017). The absorption peak at 665 cm^-1^ was due to a combination of rocking and waging vibrations of -CF_2_ groups (Tingting et al., 2005). The IR results also indicated that poly(DOPAm-*co*-PFOEA) was introduced on the surface of BPC.

### 3.3 SEM analysis

**FIGURE 4:**
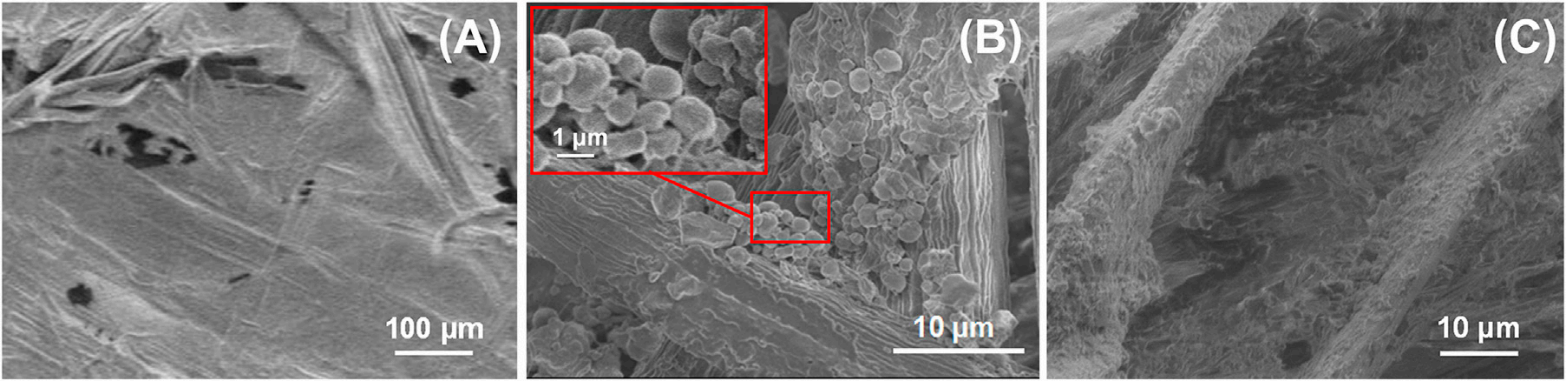
SEM images of **(A)** BPC, **(B)** BPC-TiO_2_, and **(C)** BPC-TiO_2_-F.

SEM images of the micromorphology of BPC, BPC-TiO_2_, and BPC-TiO_2_-F surfaces are shown in Figure 4. BF was embedded in PP (Figure 4A), and some fibers protruded from the BPC surface. After being treated with titanyl oxysulfate, TiO_2_ particles in the diameter range of 0.3–1 μm were formed on the surface of the BPC (Figure 4B). The BPC surface formed a micro/nanostructure after modification, and the recesses of this structure can trap air. When a liquid was dropped onto the surface of BPC-TiO_2_-F, the air in the micro/nano structure acted as a buffer that reduced the contact area between the liquid and the solid, thereby achieving superhydrophobicity (Ma et al., 2012; Bayer, 2017).

### 3.4 Wettability

**FIGURE 5:**
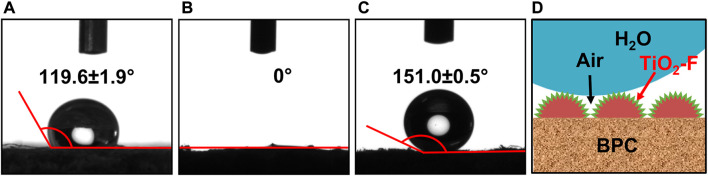
Water contact angle of **(A)** BPC, **(B)** BPC-TiO_2,_ and **(C)** BPC-TiO_2_-F. **(D)** Scheme of a water drop on the surface of BPC-TiO_2_-F in a combined Wenzel–Cassie model.

As shown in Figure 5, the wettability of BPC, BPC-TiO_2_, and BPC-TiO_2_-F was characterized by water contact angle measurements. The contact angle of BPC was 119.6° ± 1.9° (Figure 5A), and this value decreased to 0° after being modified with TiO_2_ particles (Figure 5B). This indicated that the introduction of TiO_2_ particles increased the hydrophilicity, which might be due to the increased number of hydroxyl groups. When BPC-TiO_2_ was modified by poly(DOPAm-*co*-PFOEA), the contact angle reached 151.0° ± 0.5° (Figure 5C), indicating that poly(DOPAm-*co*-PFOEA) was introduced onto BPC-TiO_2_ to produce a superhydrophobic surface.

### 3.5 Self-cleaning property

**FIGURE 6:**
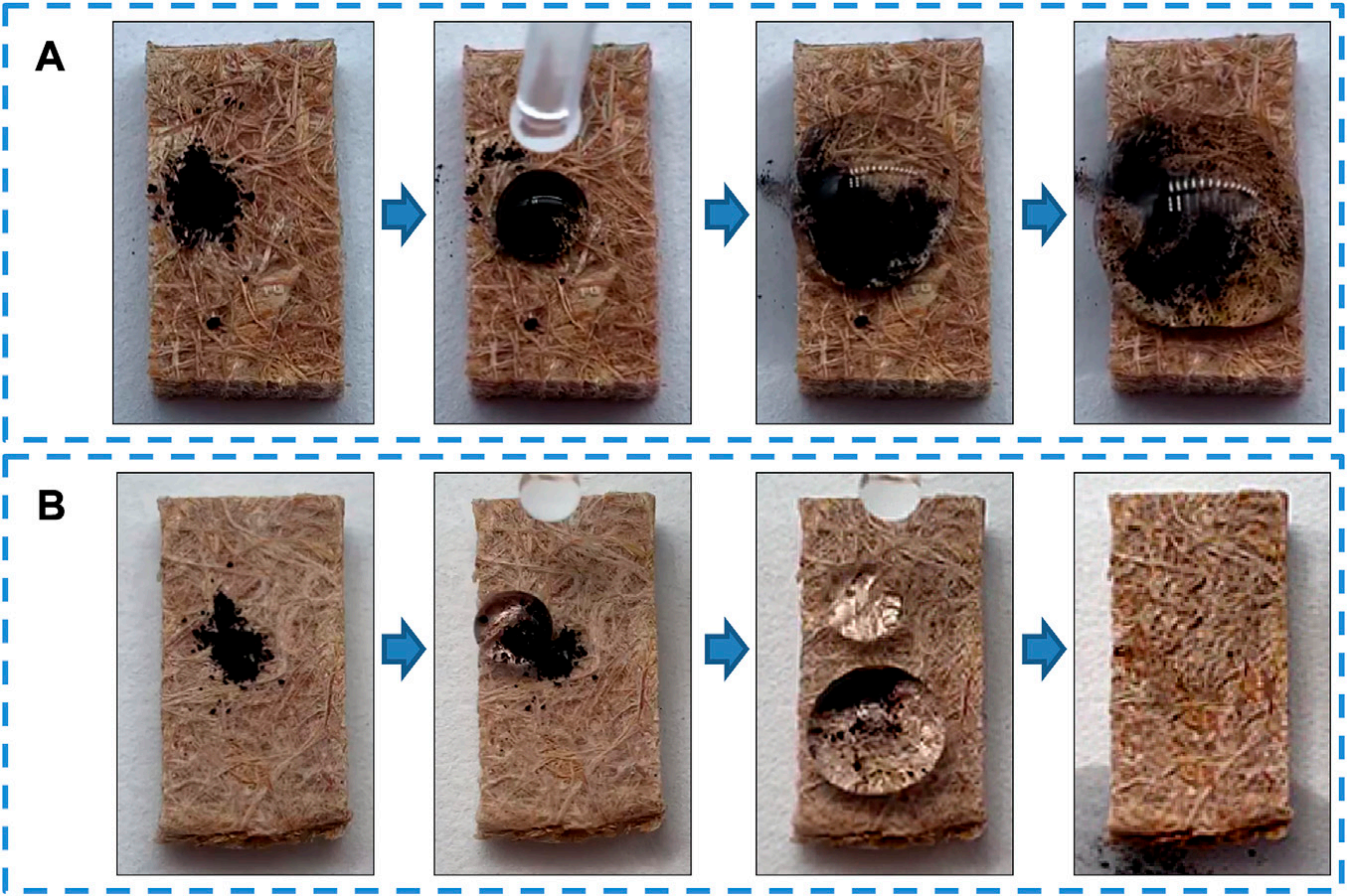
Dust removal test of **(A)** BPC and **(B)** BPC-TiO_2_-F.

Self-cleaning is an important property of superhydrophobicity and can indicate the anti-fouling ability of BPC surfaces. The self-cleaning behavior of BPC and BPC-TiO_2_-F was characterized by a dirt-removal test using Fe_3_O_4_ as a contaminant (Baidya et al., 2017), as shown in Figure 6. Black Fe_3_O_4_ was sprinkled on the surface of BPC and BPC-TiO_2_-F and then rinsed with water. For the unmodified BPC, the water droplet wrapped the black powder and adhered to the BPC surface (Figure 6A). The contact angle of the unmodified BPC was less than 150°, so the water droplets showed good wettability to the surface and spread. In contrast, the powder on the modified BPC was rapidly removed by water, and no powder residue was observed after being rinsed, as shown in Figure 6B. The above phenomena show the self-cleaning behavior of BPC-TiO_2_-F. The combined Wenzel-Cassie model (Marmur, 2004; Ma et al., 2012; Peng et al., 2022) (Figure 5D) is a reasonable explanation for the self-cleaning property. The surface with a micro/nano-hierarchical roughness and low surface energy was formed on the BPC after modification. The concave-convex on the rough surface trapped air well, decreasing contact between water droplets and modified BPC, which endowed the modified BPC with a remarkable self-cleaning capability.

### 3.6 Anti-mold effect

**FIGURE 7:**
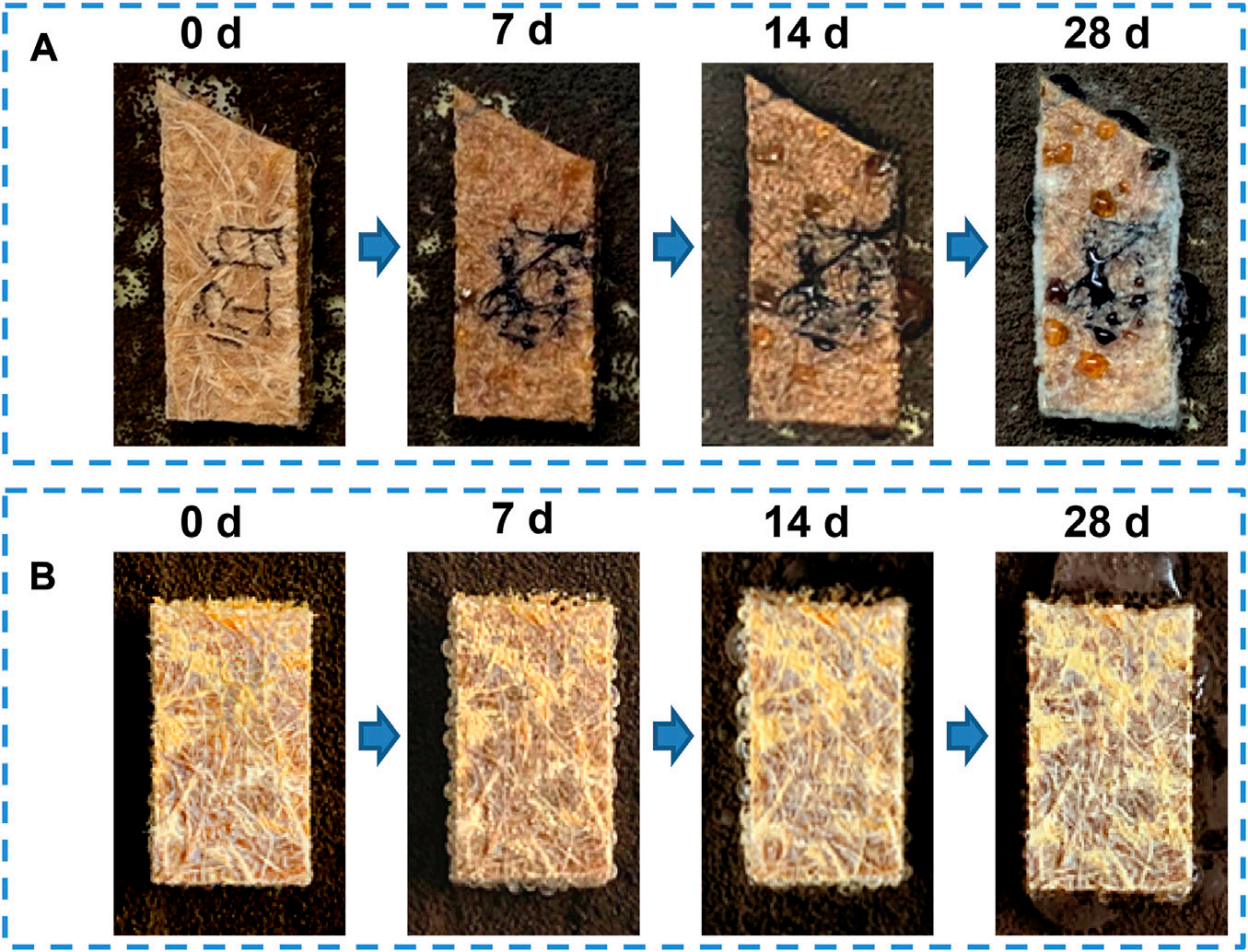
Anti-mold effect of **(A)** BPC and **(B)** BPC-TiO_2_-F.

It is essential to evaluate the effect of mold on BPC products because BF is susceptible to mold attack (Xu et al., 2013). *Aspergillus niger* is a mold that is commonly used to test the anti-mold capacity because it can quickly grow on a starch-based medium (Wang et al., 2020; Liu et al., 2022; Peng et al., 2022). Therefore, it was chosen in the anti-mold experiment, as shown in Figure 7.

Because of the presence of hydrophilic BF, the surface of BPC could be signed with water-soluble black ink (Figure 7A), while the surface of BPC-TiO_2_-F could not, indicating its superhydrophobicity (Figure 7B). After 7 days of cultivation, the marked ink became blurry, indicating that the surface of the unmodified BPC was hydrophilic, and water vapor wetted the ink during the mold culture process. As expected, white hyphae of molds were observed on the 14th day, showing that the hyphae invaded the unmodified BPC. The surface of the BPC was completely covered by mold on the 28th day, showing that the BPC had weak anti-mold activity. The unmodified BPC was easily attacked by mold fungi due to hygroscopic hydroxyl groups on the surface of BF, which provided essential conditions for mold growth (Feng et al., 2019). On the contrary, no mold was observed on the surface of the BPC-TiO_2_-F after 28 days of cultivation, indicating that the superhydrophobic surface kept the BPC-TiO_2_-F in a relatively dry state because the water droplet could not remain on its surface. This prevented mold colonies from forming. Thus, the growth of mold on the surface of BPC-TiO_2_-F was prevented.

### 3.7 Mechanical abrasion resistance

**FIGURE 8:**
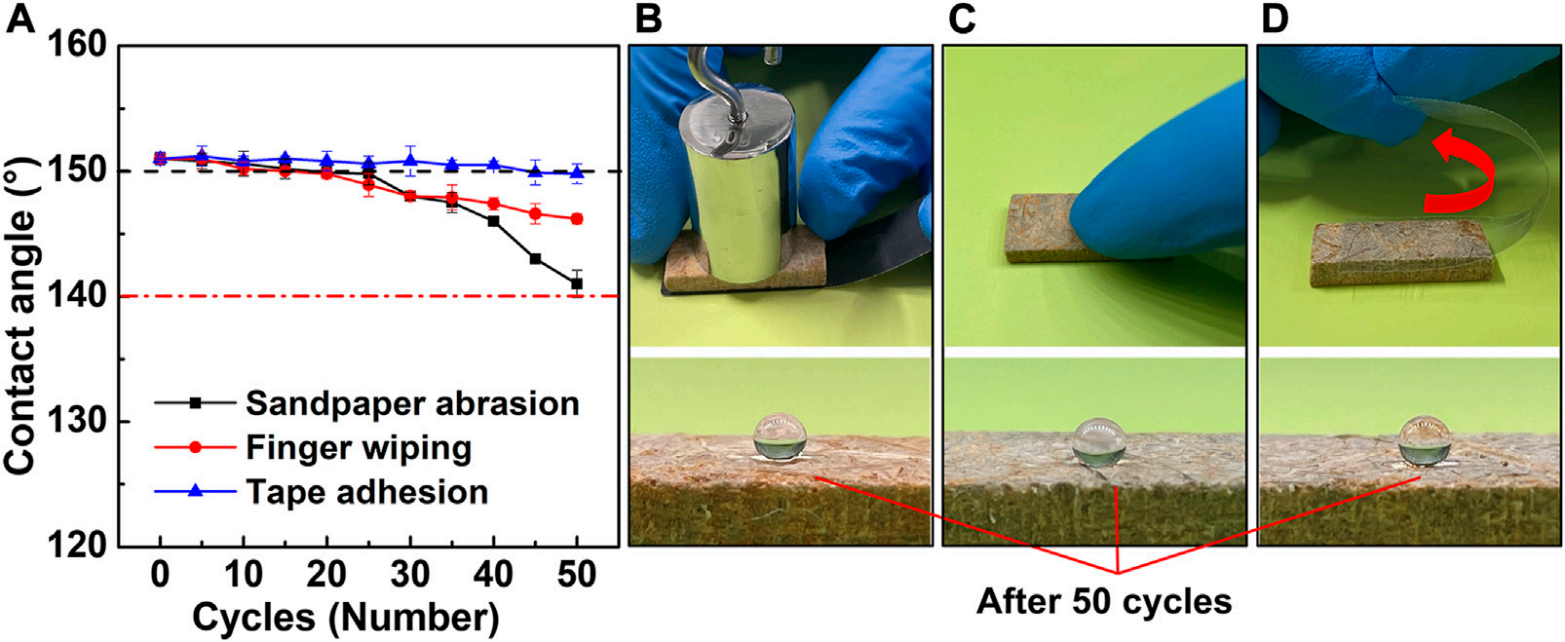
Contact angle variation of **(A)** BPC-TiO_2_-F surfaces during **(B)** sandpaper abrasion, **(C)** finger wiping, and **(D)** tape adhesion.

Mechanical durability is a critical requirement for the practical applications of BPC-TiO_2_-F. In this study, the mechanical durability of BPC-TiO_2_-F was characterized by sandpaper abrasion, tape adhesion, and finger wiping (Wang et al., 2020; Zhou et al., 2022). Changes in the water CA were recorded after every five abrasion cycles, as shown in Figure 8A. In Figure 8B, BPC-TiO_2_-F was tested against sandpaper abrasion and was loaded with a weight of 50 g. After 20 abrasion cycles, the water CA remained greater than 150°, displaying excellent abrasion resistance. After 50 abrasion cycles, the water CA dropped to 141.0°. For finger wiping (Figure 8C), the water CA dropped to 149.8° and 146.2° after 20 and 50 abrasion cycles, respectively. For tape adhesion (Figure 8D), the tape had difficulty attaching to the surface of BPC-TiO_2_-F due to its superhydrophobicity. After 40 abrasion cycles, the water CA remained higher than 150°. Although the superhydrophobic BPC-TiO_2_-F surface was obtained by a physical impregnation method, the hydrophobic layer was fixed *via* hydrogen bonding and chelation, which are physical forces. Therefore, BPC-TiO_2_-F retained its strong mechanical durability.

## 4 Conclusion

In summary, a superhydrophobic BPC with self-cleaning and anti-mold properties was fabricated. TiO_2_ particles were *in situ* loaded onto BPC *via* hydrogen bonding. Through catechol-Ti complexation, poly(DOPAm-*co*-PFOEA) with a low surface energy was deposited on the TiO_2_ particles, forming rough micro/nanostructures on the BPC surface. The contact angle of BPC-TiO_2_-F reached 151.0° ± 0.5°, indicating a superhydrophobic surface. The modified superhydrophobic BPC had good self-cleaning performance, which protected contaminants. BPC-TiO_2_-F exhibited superior anti-mold properties, and no mold colonies appeared on the surface of BPC-TiO_2_-F. Superhydrophobic BPC-TiO_2_-F could withstand a 50 g load of sandpaper and finger wiping for 20 cycles and tape adhesion abrasion for 40 cycles. BPC-TiO_2_-F, with self-cleaning, anti-mildew, and mechanical durability properties shows excellent potential for applications in automotive upholstery and building decorations.

## Data Availability

The original contributions presented in the study are included in the article/supplementary material, further inquiries can be directed to the corresponding authors.

## References

[B1] Baidya A. Ganayee M. A. Ravindran S. J. Tam K. C. Das S. K. Ras R. H. A. (2017). Organic solvent-free fabrication of durable and multifunctional superhydrophobic paper from waterborne fluorinated cellulose nanofiber building blocks. ACS Nano 11, 11091–11099. 10.1021/acsnano.7b05170 29059514

[B2] Bayer I. S. (2017). On the durability and wear resistance of transparent superhydrophobic coatings. Coatings 7, 12. 10.3390/coatings7010012

[B3] Bengtsson M. Stark N. M. Oksman K. (2007). Durability and mechanical properties of silane cross-linked wood thermoplastic composites. Compos. Sci. Technol. 67, 2728–2738. 10.1016/j.compscitech.2007.02.006

[B4] Budunoglu H. Yildirim A. Guler M. O. Bayindir M. (2011). Highly transparent, flexible, and thermally stable superhydrophobic ORMOSIL aerogel thin films. Acs Appl. Mater. Interfaces 3, 539–545. 10.1021/am101116b 21226471

[B5] Durmaz S. Erdil Y. Z. Ozgenc O. (2021). Accelerated weathering performance of wood-plastic composites reinforced with carbon and glass fibre-woven fabrics. Color. Technol. 00, 71–81. 10.1111/cote.12572

[B6] Ellinas K. Tserepi A. Gogolides E. (2011). From superamphiphobic to amphiphilic polymeric surfaces with ordered hierarchical roughness fabricated with colloidal lithography and plasma nanotexturing. Langmuir 27, 3960–3969. 10.1021/la104481p 21351799

[B7] Fei P. Xiong H. Cai J. Liu C. Yu Y. (2016). Enhanced the weatherability of bamboo fiber-based outdoor building decoration materials by rutile nano-TiO_2_ . Constr. Build. Mater. 114, 307–316. 10.1016/j.conbuildmat.2016.03.166

[B8] Feng J. Dong P. Li R. Li C. Xie X. Shi Q. (2019). Effects of wood fiber properties on mold resistance of wood polypropylene composites. Int. Biodeterior. Biodegrad. 140, 152–159. 10.1016/j.ibiod.2019.04.005

[B9] Guo J. Cao M. Ren W. Wang H. Yu Y. (2021). Mechanical, dynamic mechanical and thermal properties of TiO_2_ nanoparticles treatment bamboo fiber-reinforced polypropylene composites. J. Mater. Sci. 56, 12643–12659. 10.1007/s10853-021-06100-z

[B10] Guvendiren M. Messersmith P. B. Shull K. R. (2008). Self-assembly and adhesion of DOPA-modified methacrylic triblock hydrogels. Biomacromolecules 9, 122–128. 10.1021/bm700886b 18047285 PMC3066146

[B11] Jamaludina M. A. Baharib S. A. Zakariac M. N. Karim N. A. S. (2020). The effects of alkalization on mechanical and physical properties of bamboo-polypropylene composite. Solid State Phenom. 305, 3–7. 10.4028/www.scientific.net/ssp.305.3

[B12] Kuo P.-Y. Wang S.-Y. Chen J.-H. Hsueh H.-C. Tsai M.-J. (2009). Effects of material compositions on the mechanical properties of wood–plastic composites manufactured by injection molding. Mater. Des. 30, 3489–3496. 10.1016/j.matdes.2009.03.012

[B13] Li H. Zou X. Wei H. Li Q. Gao Q. Liu Q. (2020). SiO_2_ coated on ZnO nanorod arrays with UV-durable superhydrophobicity and highly transmittance on glass. Front. Chem. 8, 101. 10.3389/fchem.2020.00101 32154217 PMC7044700

[B14] Li X. Han Y. Ling Y. Wang X. Sun R. (2015). Assembly of layered silicate loaded quaternized chitosan/reduced graphene oxide composites as efficient absorbents for double-stranded DNA. ACS Sustain. Chem. Eng. 3, 1846–1852. 10.1021/acssuschemeng.5b00404

[B15] Liese W. (1987). Research on bamboo. Wood Science&Technology 21 (3), 189–209. 10.1007/bf00351391

[B16] Lin X. Ma W. Wu H. Cao S. Huang L. Chen L. (2016). Superhydrophobic magnetic poly(DOPAm-co-PFOEA)/Fe_3_O_4_/cellulose microspheres for stable liquid marbles. Chem. Commun. 52, 1895–1898. 10.1039/c5cc08842a 26675890

[B17] Liu X. Liang G. B. Wu X. Z. Wang W. Zhu Y. Yang S. W. (2022). Improving mechanical toughness and mold resistance of bamboo/high-density polyethylene composites by pretreatment with the fungus *Trametes versicolor* . Polym. Compos. 43, 1438–1447. 10.1002/pc.26464

[B18] Lu Q. Cheng R. Jiang H. Xia S. Zhan K. Yi T. (2022). Superhydrophobic wood fabricated by epoxy/Cu_2_(OH)_3_Cl NPs/stearic acid with performance of desirable self-cleaning, anti-mold, dimensional stability, mechanical and chemical durability. Colloids Surfaces A Physicochem. Eng. Aspects 647, 129162. 10.1016/j.colsurfa.2022.129162

[B19] Lu S. Zhang X. Tang Z. Xiao H. Zhang M. Liu K. (2021). Mussel-inspired blue-light-activated cellulose-based adhesive hydrogel with fast gelation, rapid haemostasis and antibacterial property for wound healing. Chem. Eng. J. 417, 129329. 10.1016/j.cej.2021.129329

[B20] Lu Y. Sathasivam S. Song J. Crick C. R. Carmalt C. J. Parkin I. P. (2015). Robust self-cleaning surfaces that function when exposed to either air or oil. Science 347, 1132–1135. 10.1126/science.aaa0946 25745169

[B21] Lu Y. Sun Q. Li J. Liu Y. (2014). Fabrication, characterization and photocatalytic activity of TiO_2_/cellulose composite aerogel. Key Eng. Mater. 609-610, 542–546. 10.4028/www.scientific.net/kem.609-610.542

[B22] Ma W. Wu H. Higaki Y. Otsukaa H. Takahara A. (2012). A "Non-sticky" superhydrophobic surface prepared by self-assembly of fluoroalkyl phosphonic acid on a hierarchically micro/nanostructured alumina gel film. Chem. Commun. 48, 6824–6826. 10.1039/c2cc32513f 22655297

[B23] Mahadik S. A. Kavale M. S. Mukherjee S. K. Rao A. V. (2010). Transparent Superhydrophobic silica coatings on glass by sol–gel method. Appl. Surf. Sci. 257, 333–339. 10.1016/j.apsusc.2010.06.062

[B24] Marmur A. (2004). The lotus effect: Superhydrophobicity and metastability. Langmuir 20, 3517–3519. 10.1021/la036369u 15875376

[B25] Mrad H. Alix S. Migneault S. Koubaa A. Perré P. (2018). Numerical and experimental assessment of water absorption of wood-polymer composites. Measurement 115, 197–203. 10.1016/j.measurement.2017.10.011

[B26] Nguyen-Tri P. Tran H. N. Plamondon C. O. Tuduri L. Vo D.-V. N. Nanda S. (2019). Recent progress in the preparation, properties and applications of superhydrophobic nano-based coatings and surfaces: A review. Prog. Org. Coatings 132, 235–256. 10.1016/j.porgcoat.2019.03.042

[B27] Peng J. Wu L. Zhang H. Wang B. Si Y. Jin S. (2022). Research progress on eco-friendly superhydrophobic materials in environment, energy and biology. Chem. Commun. 58, 11201–11219. 10.1039/d2cc03899d 36125075

[B28] Ren D. Zhang X. Yu Z. Wang H. Yu Y. (2020). Enhancing the mechanical and water resistance performances of bamboo particle reinforced polypropylene composite through cell separation. Holzforschung 75 (3), 269–280. 10.1515/hf-2019-0289

[B29] Rowell R. M. (2007). Challenges in biomass–thermoplastic composites. J. Polym. Environ. 15, 229–235. 10.1007/s10924-007-0069-0

[B30] Sanjay M. R. Siengchin S. Parameswaranpillai J. Jawaid M. Pruncu C. I. Khan A. (2019). A comprehensive review of techniques for natural fibers as reinforcement in composites: Preparation, processing and characterization. Carbohydr. Polym. 207, 108–121. 10.1016/j.carbpol.2018.11.083 30599990

[B31] Shalbafan A. Benthien J. T. Welling J. Barbu M. C. (2013). Flat pressed wood plastic composites made of milled foam core particleboard residues. Eur. J. Wood Wood Prod. 71, 805–813. 10.1007/s00107-013-0745-9

[B32] Sobczak L. Lang R. W. Haider A. (2012). Polypropylene composites with natural fibers and wood – general mechanical property profiles. Compos. Sci. Technol. 72, 550–557. 10.1016/j.compscitech.2011.12.013

[B33] Tingting Y. Hui P. Shiyuan C. Park I. J. (2005). Surface immobilization of perfluorinated acrylate copolymers by self-crosslinking. J. Fluor. Chem. 126, 1570–1577. 10.1016/j.jfluchem.2005.09.008

[B34] Vedat Ç. Fatih M. (2020). Effect of wood particle size on selected properties of neat and recycled wood polypropylene composites. BioResources 15 (2), 3427–3442. 10.15376/biores.15.2.3427-3442

[B35] Wang C. Cai L. Shi S. Q. Wang G. Cheng H. Zhang S. (2019). Thermal and flammable properties of bamboo pulp fiber/high-density polyethylene composites: Influence of preparation Technology, nano calcium carbonate and fiber content. Renew. Energy 134, 436–445. 10.1016/j.renene.2018.09.051

[B36] Wang S. L. Liu C. Y. Liu G. C. Zhang M. Li J. Wang C. Y. (2011b). Fabrication of superhydrophobic wood surface by a sol-gel process. Appl. Surf. Sci. 258, 806–810. 10.1016/j.apsusc.2011.08.100

[B37] Wang S. Shi J. Liu C. Xie C. Wang C. (2011a). Fabrication of a superhydrophobic surface on a wood substrate. Appl. Surf. Sci. 257, 9362–9365. 10.1016/j.apsusc.2011.05.089

[B38] Wang S. Wang C. Liu C. Zhang M. Ma H. Li J. (2012). Fabrication of superhydrophobic spherical-like α-FeOOH films on the wood surface by a hydrothermal method. Colloids Surfaces A Physicochem. Eng. Aspects 403, 29–34. 10.1016/j.colsurfa.2012.03.051

[B39] Wang Y. Tang Z. Lu S. Zhang M. Liu K. Xiao H. (2020). Superhydrophobic wood grafted by poly(2-(perfluorooctyl)ethyl methacrylate) via ATRP with self-cleaning, abrasion resistance and anti-mold properties. Holzforschung 74, 799–809. 10.1515/hf-2019-0184

[B40] Windeisen E. Strobel C. Wegener G. (2007). Chemical changes during the production of thermo-treated beech wood. Wood Sci. Technol. 41, 523–536. 10.1007/s00226-007-0146-5

[B41] Wu H. Wu L. Lu S. Lin X. Xiao H. Ouyang X. (2018). Robust superhydrophobic and superoleophilic filter paper via atom transfer radical polymerization for oil/water separation. Carbohydr. Polym. 181, 419–425. 10.1016/j.carbpol.2017.08.078 29253991

[B42] Xu B. Cai Z. (2008). Fabrication of a superhydrophobic ZnO nanorod array film on cotton fabrics via a wet chemical route and hydrophobic modification. Appl. Surf. Sci. 254, 5899–5904. 10.1016/j.apsusc.2008.03.160

[B43] Xu G. Wang L. Liu J. Hu S. (2013). Decay resistance and thermal stability of bamboo preservatives prepared using camphor leaf extract. Int. Biodeterior. Biodegrad. 78, 103–107. 10.1016/j.ibiod.2012.12.001

[B44] Xu H. Nishida J. Ma W. Wu H. Kobayashi M. Otsuka H. (2012). Competition between oxidation and coordination in cross-linking of polystyrene copolymer containing catechol groups. ACS Macro Lett. 1, 457–460. 10.1021/mz200217d 35585740

[B45] Yang H. Deng Y. (2008). Preparation and physical properties of superhydrophobic papers. J. Colloid Interface Sci. 325, 588–593. 10.1016/j.jcis.2008.06.034 18603258

[B46] Yu Y. Tian G. Wang H. Fei B. Wang G. (2011). Mechanical characterization of single bamboo fibers with nanoindentation and microtensile technique. Holzforschung 65, 113–119. 10.1515/hf.2011.009

[B47] Yu Y. Wang H. Lu F. Tian G. Lin J. (2014). Bamboo fibers for composite applications: A mechanical and morphological investigation. J. Mater. Sci. 49, 2559–2566. 10.1007/s10853-013-7951-z

[B48] Zeng H. Hwang D. S. Israelachvili J. N. Waite J. H. (2010). Strong reversible Fe^3+^-mediated bridging between dopa-containing protein films in water. Proc. Natl. Acad. Sci. U. S. A. 107, 12850–12853. 10.1073/pnas.1007416107 20615994 PMC2919964

[B49] Zhang W. Yao X. Khanal S. Xu S. (2018). A novel surface treatment for bamboo flour and its effect on the dimensional stability and mechanical properties of high density polyethylene/bamboo flour composites. Constr. Build. Mater. 186, 1220–1227. 10.1016/j.conbuildmat.2018.08.003

[B50] Zhou C. Chen Q. Chen Q. Yin H. Wang S. Hu C. (2022). Preparation of TiO_2_ superhydrophobic composite coating and studies on corrosion resistance. Front. Chem. 10, 943055. 10.3389/fchem.2022.943055 35873046 PMC9304710

[B51] Zimmermann J. Reifler F. A. Fortunato G. Gerhardt L.-C. Seeger S. (2008). A simple, one step approach to durable and robust superhydrophobic textiles. Adv. Funct. Mater. 18, 3662–3669. 10.1002/adfm.200800755

